# Analysis of Estimated and Measured Glomerular Filtration Rates and the CKD-EPI Equation Race Coefficient in the Chronic Renal Insufficiency Cohort Study

**DOI:** 10.1001/jamanetworkopen.2021.17080

**Published:** 2021-07-15

**Authors:** Chi-yuan Hsu, Wei Yang, Alan S. Go, Rishi V. Parikh, Harold I. Feldman

**Affiliations:** 1 Division of Nephrology, Department of Medicine, University of California, San Francisco, San Francisco; 2 Division of Research, Kaiser Permanente Northern California, Oakland; 3 Center for Clinical Epidemiology and Biostatistics, Department of Biostatistics, Epidemiology and Informatics, Perelman School of Medicine at the University of Pennsylvania, Philadelphia; 4 Department of Health Systems Science, Kaiser Permanente Bernard J. Tyson School of Medicine, Pasadena, California; 5 Departments of Medicine, Epidemiology and Biostatistics, University of California, San Francisco, San Francisco; 6 Department of Medicine (Nephrology), Stanford University School of Medicine, Palo Alto

## Abstract

This diagnostic/prognostic study examines the estimated glomerular filtration rates (eGFR) and iothalamate clearance glomerular filtration rates (iGFR) and the Chronic Kidney Disease-Epidemiology (CKD-EPI) equation race coefficient in the Chronic Renal Insufficiency Cohort study.

## Introduction

Zelnick et al^1^ analyzed data from the Chronic Renal Insufficiency Cohort (CRIC) obtained from the National Institute of Diabetes and Digestive and Kidney Diseases central repository and reported that the Chronic Kidney Disease-Epidemiology (CKD-EPI) equation estimated glomerular filtration rates (eGFR) were higher than iothalamate clearance glomerular filtration rates (iGFR) among self-reported Black participants with iGFR less than 45 mL/min/1.73 m^2^. Using the CKD-EPI equation without a (Black vs non-Black) race coefficient appeared to attenuate the difference between eGFR and iGFR (eGFR − iGFR). The authors suggested that the latter approach should be used more widely in clinical practice.

We believe that examining potential bias in eGFR values within subgroups defined by iGFR is problematic. An unbiased eGFR estimation aims to ensure that for the set of individuals who have the same eGFR value, the mean iGFR value is the same as the eGFR. The correlation between the difference between eGFR and iGFR (eGFR − iGFR) and iGFR is expected for an unbiased eGFR estimation.^2^ We offer an alternative view of the data here with different clinical implications.

## Methods

The CRIC study was approved by participating sites’ institutional review boards.^3^ All participants provided written consent. Race/ethnicity data were self-reported. This diagnostic study followed the Transparent Reporting of a Multivariable Prediction Model for Individual Prognosis or Diagnosis (TRIPOD) reporting guideline.

We first replicated what Zelnick et al^1^ observed by conducting cross-sectional analyses of CRIC data from the University of Pennsylvania CRIC Scientific and Data Coordinating Center.^4^ For ease of interpretation, we used only baseline data. Among the subset of CRIC participants who self-identified as Black and had iGFR less than 45 mL/min/1.73 m^2^, we quantified the difference between eGFR and iGFR.

We next quantified the difference between eGFR and iGFR among Black participants with iGFR greater than or equal to 45 mL/min/1.73 m^2^, among non-Black participants with iGFR less than 45 mL/min/1.73 m^2^, and among Black participants with eGFR less than 45 mL/min/1.73 m^2^. The analysis was stratified by Black and non-Black participants because this was the way the race coefficient in the CKD-EPI equation was dichotomized.

Statistical analysis was performed using R software version 4.0 (R Project for Statistical Computing) from February to March 2021. For details about the theory for why (eGFR − iGFR) is higher when iGFR is lower, see the eAppendix in the Supplement.

## Results

Among 1422 study participants, 619 (43.5%) were women, 584 (41.1%) self-identified as non-Hispanic White, 525 (36.9%) as non-Hispanic Black, 214 (15.0%) as Hispanic, and 99 (7.0%) as various other races/ethnicities. The mean (SD) age was 56.0 (12.3) years.

Similar to Zelnick et al,^1^ we noted apparent overestimation of iGFR by eGFR among Black participants with iGFR less than 45 mL/min/1.73 m^2^ (ie, eGFR higher than iGFR by a mean value of 4.0 mL/min/1.73 m^2^ [95% CI, 2.9 to 5.0 mL/min/1.73 m^2^]) (Table) (Figure A). This overestimation was not seen among Black participants with iGFR greater than or equal to 45 mL/min/1.73 m^2^ (Table) (Figure A).

**Table.** Distribution of iGFR, CKD-EPI eGFR Differences in CRIC Participants Stratified by iGFR vs eGFR and Self-Reported Race Categories

Variable	Race	No.	Mean (SD), mL/min/1.73 m^2^		eGFR-iGFR, mean (95% CI), mL/min/1.73 m^2^
			iGFR	eGFR	
**iGFR, mL/min/1.73 m^2^**					
<45	Black	269	31.9 (8.5)	35.9 (10.7)	4.0 (2.9 to 5.0)
	Not Black	420	31.9 (8.1)	35.2 (9.4)	3.2 (2.5 to 3.9)
≥45	Black	259	62.8 (13.9)	57.7 (12.9)	−5.2 (−6.5 to −3.8)
	Not Black	474	63.1 (16.2)	56.0 (13.1)	−7.2 (−8.2 to −6.1)
**eGFR, mL/min/1.73 m^2^**					
<45	Black	258	34.2 (12.1)	33.5 (7.6)	−0.7 (−1.8 to 0.4)
	Not Black	453	35.1 (11.3)	34.0 (7.3)	−1.1 (−1.9 to −0.3)
≥45	Black	270	59.3 (16.7)	59.0 (11.5)	−0.3 (−1.8 to 1.2)
	Not Black	441	62.2 (18.3)	58.6 (11.2)	−3.5 (−4.8 to −2.3)

Abbreviations: CKD-EPI, Chronic Kidney Disease-Epidemiology; CRIC, Chronic Renal Insufficiency Cohort; eGFR, estimated glomerular filtration rate; iGFR, iothalamate clearance-based glomerular filtration rate.

**Figure.**
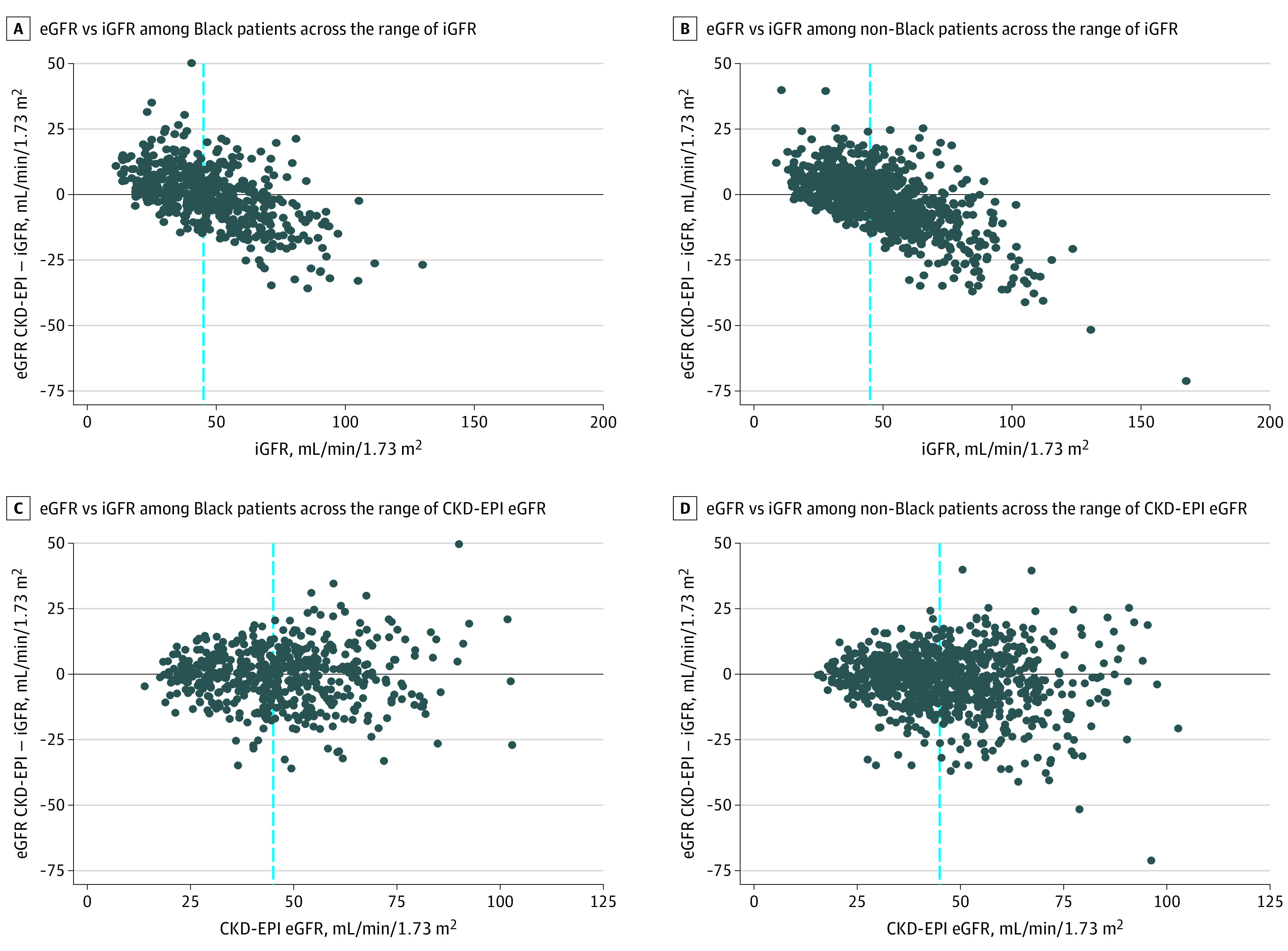
Difference in Estimated Glomerular Filtration Rate (eGFR) vs Iothalamate Clearance-Based Glomerular Filtration Rate (iGFR) Stratified by Self-Reported Race The difference in eGFR vs iGFR across the range of iGFR (A and B) or CKD-EPI eGFR (C and D) stratified by self-reported race. The vertical dotted line represents an eGFR/iGFR of 45 mL/min/1.73 m^2^.

Among non-Black participants with baseline iGFR less than 45 mL/min/1.73 m^2^, there was also apparent overestimation of iGFR by eGFR (eGFR higher than iGFR by a mean value of 3.2 mL/min/1.73 m^2^ [95% CI, 2.5 to 3.9 mL/min/1.73 m^2^]) (Table) (Figure B).

Among Black participants with eGFR less than 45 mL/min/1.73 m^2^, their mean eGFR did not overestimate iGFR (Table) (difference between eGFR and iGFR was −0.7 mL/min/1.73 m^2^ [95% CI, −1.8 to 0.4 mL/min/1.73 m^2^]) (Table) (Figure C). Results in other subgroups are shown in the Table and the Figure.

## Discussion

Among CRIC participants with eGFR less than 45 mL/min/1.73 m^2^, there was no observed bias in the CKD-EPI eGFR equation that included a coefficient for race among Black participants. This study’s findings suggest that removing the race coefficient from the current CKD-EPI equation would introduce bias rather than improve its accuracy. These findings contrast with those of Zelnick et al,^1^ whose observation of greater accuracy in the performance of the CKD-EPI equation among the subset of Black CRIC participants with iGFR less than 45 mL/min/1.73 m^2^ after removal of the race coefficient was a consequence of studying subgroups formed using the values of the quantity being estimated (ie, iGFR).^2^

Importantly, the same apparent overestimation of iGFR by CKD-EPI eGFR was seen among non-Black CRIC participants selected on the basis of iGFR less than 45 mL/min/1.73 m^2^, highlighting that the bias observed was neither correlated to race nor the use of an estimating equation incorporating a race coefficient, but rather subgroup stratification based on iGFR.

Our study was limited by inclusion of only research volunteers from the US. It also did not have robust representation of Asian individuals and other racial/ethnic subgroups.

Based on this study’s findings, we do not believe CRIC data support the notion that dropping the race coefficient in the current CKD-EPI equation enhances accuracy in kidney function estimation. We do support the long-term goal of minimizing need to consider race in estimating GFR and in other clinical algorithms.^5,6^

## Notes

Supplement. **eAppendix.** Theory Behind the Reason (eGFR − iGFR) is Higher When iGFR is Lower
