# Supplementary material for: Analysis of Estimated and Measured Glomerular Filtration Rates and the CKD-EPI Equation Race Coefficient in the Chronic Renal Insufficiency Cohort Study
Source: JAMA Netw Open. 2021 Jul 15;4(7):e2117080. doi: 10.1001/jamanetworkopen.2021.17080 (PMC8283556; doi:10.1001/jamanetworkopen.2021.17080)
Supplement: Supplement. — eAppendix. Theory Behind the Reason (eGFR − iGFR) is Higher When iGFR is Lower [file jamanetwopen-e2117080-s001.pdf]

Supplemental Online Content

Hsu CY, Yang W, Go AS, Parikh RV, Feldman HI. Analysis of estimated and measured glomerular filtration rates and the CKD-EPI equation race coefficient in the Chronic Renal Insufficiency Cohort study. *JAMA Netw Open.* 2021;4(7):e2117080. doi:10.1001/jamanetworkopen.2021.17080

eAppendix. Theory Behind the Reason (eGFR − iGFR) is Higher When iGFR is Lower

This supplemental material has been provided by the authors to give readers additional information about their work.

© 2021 Hsu CY et al. *JAMA Network Open.*

eAppendix. Theory Behind the Reason (eGFR − iGFR) is Higher When iGFR is Lower

CKD-EPI type estimating equations are derived by regressing iGFR on serum creatinine and other predictors such as demographic variables. An unbiased eGFR estimation should ensure that for the set of individuals who have the same eGFR value, the average iGFR value is the same as eGFR. But for each individual person, his/her eGFR value does not perfectly match his/her iGFR value. In short, there is residual (ε), defined as the difference between the observed outcome Y (e.g., iGFR) and predicted outcome $\hat{Y}$ (e.g., eGFR), i.e., $\varepsilon = Y - \hat{Y}$. Note that the difference between eGFR and iGFR is the negative residual, i.e., $-\varepsilon$. The correlation between ε and Y , i.e., $Cor(\varepsilon, Y)$ by definition equals

$$Cor(\varepsilon, Y) = \frac{Cov(\varepsilon, Y)}{\sqrt{Var(\varepsilon)}\sqrt{Var(Y)}} = \frac{Cov(\varepsilon, \hat{Y} + \varepsilon)}{\sqrt{Var(\varepsilon)}\sqrt{Var(Y)}} = \frac{Var(\varepsilon) + Cov(\varepsilon, \hat{Y})}{\sqrt{Var(\varepsilon)}\sqrt{Var(Y)}}$$

where $Cov(.,.)$ denotes the covariance between two random variables and $Var(.)$ denotes the variance of a random variable. For an unbiased equation, the residual ε is independent of the predicted outcome $\hat{Y}$, so that $Cov(\varepsilon, \hat{Y}) = 0$. Consequently, the correlation between ε and Y equals $\frac{\sqrt{Var(\varepsilon)}}{\sqrt{Var(Y)}}$, which is positive unless the variance of the residual is 0 (which is never the case in reality since eGFR never perfectly matches iGFR at an individual person level). Thus, a negative correlation between (eGFR-iGFR) and iGFR is expected and the average (eGFR-iGFR) tends to be positive at lower iGFR values.

© 2021 Hsu CY et al. *JAMA Network Open.*
